# Pentraxin 3, TNF-α, and LDL-C Are Associated With Carotid Artery Stenosis in Patients With Ischemic Stroke

**DOI:** 10.3389/fneur.2019.01365

**Published:** 2020-01-10

**Authors:** Li Yi, Jie Tang, Cuige Shi, Tingting Zhang, Jing Li, Fang Guo, Wei Zhang

**Affiliations:** ^1^Department of Neurology, Beijing Friendship Hospital, Capital Medical University, Beijing, China; ^2^Department of Neurosurgery, Xuanwu Hospital, Capital Medical University, Beijing, China; ^3^National Research Institute for Family Planning, Beijing, China; ^4^Department of Radiology, Beijing Friendship Hospital, Capital Medical University, Beijing, China

**Keywords:** Pentraxin 3 (PTX3), carotid artery stenosis (CAS), TNF-α, LDL-cholesterol, ischemic stroke

## Abstract

**Background:** Carotid artery stenosis (CAS), typically resulting from atherosclerotic progression, is more common than intracranial atherosclerotic disease in patients with cerebrovascular disease. Prior literature has incompletely established the relationship of Pentraxin 3 (PTX3) and CAS complexity and severity. This study aims to more thoroughly evaluate the association of plasma PTX3 levels and the prevalence and severity of CAS in patients with cerebrovascular disease.

**Methods:** Two hundred and six patients with ischemic stroke underwent multiphase computerized tomography angiography (CTA) of the head and neck to assess the presence and severity of carotid artery stenosis. Patients were divided into groups with either no carotid artery stenosis (CAS-free) or carotid artery stenosis (CAS). The CAS group was further divided into groups based on the degree of stenosis and the number of involved vessels. The PTX3 and Tumor Necrosis Factor-alpha (TNF-α) concentration were measured by ELISA.

**Results:** Plasma levels of PTX3, TNF-α, and low-density lipoprotein cholesterol (LDL-C) were increased significantly in the CAS group patients vs. the CAS-free group (*p* = 0.000, 0.002, 0.002, respectively). Within the CAS group, PTX3, TNF-α, and LDL-C were significantly elevated in stenosis of ≥50% group compared to <50% group (*p* = 0.001, 0.002, 0.049, respectively). The multivariate logistic binary regression analysis revealed that increased age, elevated levels of PTX3, LDL-C, and TNF-α were all independent risk factors for occurrence of carotid stenosis. PTX3 level correlated with the severity of carotid stenosis.

**Conclusions:** High plasma PTX3 levels, TNF-α, and LDL-C are significantly correlated with the prevalence and severity of carotid artery stenosis. PTX3 may be a more powerful predictor for the severity of carotid artery stenosis.

## Introduction

Cerebrovascular disease is becoming increasingly prevalent throughout the world and is a significant cause of morbidity and mortality globally. Prior reports estimate that ~15% of ischemic strokes are caused by large vessel cerebrovascular diseases (1). Carotid artery stenosis (CAS), typically resulting from atherosclerotic progression, is more common than intracranial atherosclerotic disease in patients with cerebrovascular disease, and is one of the major causes of stroke and transient ischemic attack (TIA) (2–4). Recently, the role of inflammation in these processes has been recognized as an important risk factor, with many studies focusing on the relationship between inflammatory markers and cerebrovascular disease.

Pentraxin 3 (PTX3) is a member of the pentraxin superfamily which play a role in acute immune responses and are released by many cell types, including endothelial cells, smooth muscle cells, and mononuclear phagocytes. PTX3 is not directly secreted from blood vessels on to lesions in ongoing vascular inflammation (5). Recent work suggests that PTX3 is more specific for the development of atherosclerosis than other markers such as hs-CRP (6, 7). Several recent studies have also suggested that PTX3 is a useful biomarker for coronary artery disease and heart failure. In these conditions, as well as metabolic syndrome, levels of PTX3 may also correlate with the severity of the disease (8).

Prior literature regarding the association of PTX3 in cerebrovascular disease has incompletely established the relationship of PTX3 and CAS complexity and severity. This study aims to more thoroughly evaluate the association of plasma PTX3 levels and the incidence and severity of CAS in patients with ischemic stroke.

## Materials and Methods

### Patient Selection Method

All patients were prospectively recruited from the Department of Neurology at Friendship Hospital of Capital Medical University in Beijing, China and met the criteria of the 2010 Chinese guidelines for diagnosis and treatment of acute ischemic stroke. Patients were interviewed either in clinic or the inpatient department and all signed the informed consent form. Our study followed the principles of the Declaration of Helsinki and was approved by the Bioethics Committee of Beijing Friendship Hospital, Capital Medical University (2019-P2-063-01).

Inclusion criteria for this study included patients with ischemic stroke, no recent use of anti-inflammatory medication within the last 2 weeks, and a complete medical record. Exclusion criteria included patients with prior clinical diagnoses of acute ischemic stroke, acute coronary syndrome, periphary artery disease, heart failure, renal failure, hepatic failure, drug addiction/abuse, peripheral artery disease, infection, and chronic or systemic inflammatory diseases.

### Patient Classification

A total of 249 patients were enrolled in the study initially. According to the exclusion criteria, 43 patients were excluded. Finally 206 patients were included in this study for further analysis. All participants were clinically stable based on their medical records, clinical symptoms, and brain MRI testing.

All patients underwent multiphase computerized tomography angiography (CTA) of the head and neck to assess the presence and severity of both common and internal carotid artery stenosis by revolution CT (GE, USA). The captured head and neck CTA images were uploaded to Advantage Workstation 4.6 and reconstructed by single-layer MPR. Carotid artery stenosis was quantitatively evaluated by using the North American Symptomatic Carotid Endarterectomy Trial (NASCET) system (9). The luminal diameter at the point of greatest stenosis and the normal part of the artery beyond the carotid bulb were measured by the principal neuroradiologist on the angiograms of each patient. The percent stenosis was determined by calculating the ratio of these two measurements, with use of the view showing the greatest degree of narrowing. The stenosis were assessed by area manually by two experienced radiologists independently. The most severe stenosis part were recorded for analysis.

We divided patients into two groups with either no carotid artery stenosis (CAS-free) or carotid artery stenosis (CAS). The CAS group was further divided into two groups based on severity of CAS. CAS of ≥50% was classified as severe stenosis (SS), while the stenosis of <50% was classified as mild stenosis (MS). Final patient classification was done using the degree of stenosis and the number of involved vessels, creating a total of 5 groups. These included groups with no stenosis, unilateral mild (<50%), bilateral mild (<50%), unilateral severe (≥50%), and bilateral severe (≥50%) stenosis, representing groups number 1 through 5, respectively.

Blood sample were collected prior to the CTA test and within 48 h after ischemic stroke attack, we used an Olympus 5800 automated chemistry analyzer (Olympus, Tokyo, Japan) to conduct routine blood biochemistry tests assessing total cholesterol (CHOL), low-density lipoprotein cholesterol (LDL-C), high density lipoprotein cholesterol (HDL-C), triglyceride (TG), creatinine (Cr), aspartate aminotransferase (AST), and alanine aminotransferase (ALT).

We also use the median concentration of PTX3 (5.90 ng/ml) to further subdivide patients into PTX3 high (≥5.90 ng/ml) and low (<5.90 ng/ml) groups.

### PTX3 and Tumor Necrosis Factor-alpha (TNF-α) Measurement Methods

We collected blood samples after 12 h fasting on the day of carotid artery angiography. Plasma was extracted in EDTA tubes and separated by centrifugation at 3,500 rpm for 10 min. The supernatant containing the plasma was collected and stored in −80°C until the assays were performed. The PTX3 concentration was measured by using a commercial human ELISA kit (Quantakine DPTX 30, R&D system, UK) and TNF-α measurement was done using another ELISA kit (BD-Biosciences, San Jose, CA, USA).

### Statistical Analysis

For continuous variables, independent *t*-tests were used when patients were divided into two groups, and we used ANOVA tests when analyzing more than 2 groups. A chi-square test was used for categorical data. Logistic regression analysis was used to study risk factors for the presence of carotid stenosis (CAS vs. CAS-free) and severity of stenosis (SS vs. MS). Linear regression was performed to compare PTX3 levels with the severity of carotid stenosis, using 5 groups classified by degree of stenosis and the number of involved vessels, as described earlier. We performed multivariate linear regression to study the relationship between PTX3 level and age, gender, smoking history, cerebrovascular disease family history, hypertension, diabetes mellitus, history of statin use, BMI, TNF-α level, Chol, TG, HDL, and hs-CRP. All statistical analysis was performed using IBM SPSS statistics 23.

## Results

### Patient Demographics Based on CAS

Compairson between groups was performed using independent *t*-tests or ANOVA tests where appropriate. *P* < 0.05 was considered significant.

One hundred and seven of the 206 patients had CAS, with 50 patients (46.73%) having stenosis ≥50% (SS) and 57 patients (53.27%) having mild stenosis (MS). Within the SS group, 34 patients (31.78%) had unilateral arterial disease and16 patients (14.95%) had bilateral arterial disease. In the MS group, 33 (30.84%) patients had single-vessel disease, while 24 (22.43%) patients had two-vessel disease.

Table 1 shows the clinical and laboratory tests of the patients in the CAS and CAS-free groups. No significant differences were found in gender, age, smoking, hypertension, diabetes mellitus, history of statin use, BMI, CHOL, TG, and hs-CRP between the CAS and CAS-free groups. Plasma levels of LDL-C, TNF-α, and PTX3 were increased significantly in the CAS group patients vs. the CAS-free group (*p* = 0.002, 0.002, 0.000, respectively), and family history of cerebrovascular disease was significantly more common in the CAS group (*p* = 0.005) (Table 1).

**Table 1:** Biochemical characteristics of CAS and CAS-free groups.

**Parameters**	**CAS**	**CAS-free**	**t/x^**2**^**	***p*-value**
	***N* = 107**	***N =* 99**		
Gender, male (%)	62.6	59.6	0.198	0.670
Age, years	64.10 ± 7.70	62.30 ± 9.98	1.455	0.147
Family history of cerebrovascular disease (%)	51.4	31.3	8.533	0.005^*^
Smoking (%)	49.5	36.4	3.634	0.068
Hypertension (%)	64.5	56.6	1.352	0.257
Diabetes mellitus (%)	57.9	56.4	0.034	0.662^*^
History of statin use (%)	39.2	28.3	2.758	0.107
BMI, kg/m^2^	27.58 ± 2.91	26.93 ± 3.20	1.518	0.131
CHOL, mmol/l	6.10 ± 0.62	6.07 ± 1.03	0.217	0.829
LDL-C, mmol/l	3.13 ± 0.75	2.45 ± 0.51	5.632	0.002^*^
TG, mmol/l	1.87 ± 0.88	1.80 ± 0.68	0.567	0.572
HDL-C, mmol/l	1.18 ± 0.31	1.19 ± 0.62	−0.152	0.877
hs-CRP, mg/l	3.01 ± 1.06	2.9 ± 1.12	0.720	0.472
TNF-α, ng/ml	1.51 ± 0.23	1.31 ± 0.19	5.031	0.002^*^
PTX3, ng/ml	7.95 ± 2.76	4.84 ± 1.42	10.267	<0.001^*^

*BMI, body mass index; CHOL, total cholesterol; LDL-C, low-density lipoprotein cholesterol; TG, triglyceride; HDL-C, high density lipoprotein cholesterol; hs-CRP, hypersensitivity C-reactive protein; TNF-α, tumor necrosis factor-alpha; PTX3, pentraxin 3*.

* *P < 0.05*.

Within the CAS group, LDL-C, TNF-α, and PTX3 were significantly elevated in the SS group (*p* = 0.049, 0.002, 0.001, respectively) compared to the MS group, however no statistical difference infamily history of cerebrovascular disease was noted between these two groups (*p* = 1.000) (Table 2).

**Table 2:** Biochemical characteristics of severe (SS) and mild stenosis (MS) patients within the CAS group.

**Parameters**	**SS**	**MS**	**t/x^**2**^**	***p*-value**
	***N* = 50**	***N* = 57**		
Gender, male (%)	62.0	63.2	0.015	1.000
Age, years	64.12 ± 7.59	64.09 ± 7.86	0.022	0.983
Family history of cerebrovascular disease (%)	52.0	50.9	0.013	1.000
Smoking (%)	52.0	47.4	0.229	0.700
Hypertension (%)	68.0	61.4	0.506	0.546
Diabetes mellitus (%)	62.0	54.4	0.634	0.441
History of statin use (%)	44.0	35.1	0.887	0.428
BMI, kg/m^2^	28.04 ± 2.75	27.18 ± 3.00	1.541	0.126
CHOL, mmol/l	6.11 ± 0.62	6.09 ± 0.62	0.123	0.903
LDL-C, mmol/l	3.28 ± 0.73	3.00 ± 0.74	1.998	0.049^*^
TG, mmol/l	1.87 ± 0.83	1.87 ± 0.92	0.001	0.999
HDL-C, mmol/l	1.19 ± 0.35	1.18 ± 0.26	0.119	0.905
hs-CRP, mg/l	3.10 ± 1.14	2.94 ± 0.99	0.773	0.441
TNF-α, pg/ml	1.63 ± 0.22	1.40 ± 0.17	5.889	0.002^*^
PTX3, ng/ml	9.56 ± 2.99	6.53 ± 1.50	6.459	0.001^*^

*BMI, body mass index; CHOL, total cholesterol; LDL-C, low-density lipoprotein cholesterol; TG, triglyceride; HDL-C, high density lipoprotein cholesterol; hs-CRP, hypersensitivity C-reactive protein; TNF-α, tumor necrosis factor-alpha; PTX3, pentraxin 3*.

* *P < 0.05*.

In both two tables, PTX3 is significantly higher in the CAS group than in the CAS-free group (7.95 ± 2.76 ng/ml vs. 4.84 ± 1.42 ng/ml, *P* < 0.001) (Table 1). PTX3 was also significantly higher in the SS group vs. the MS group (9.56 ± 2.99 ng/ml vs. 6.53 ± 1.50 ng/ml, *p* = 0.001) (Table 2).

### Patient Demographics Based on PTX3

The levels of LDL-C and TNF-α were significantly higher in the PTX3 high group (*p* = 0.013, *p* = 0.018, respectively), but no significant differences were observed in plasma HDL-C, CHOL, TG, family history of cerebrovascular disease, and BMI between the two groups (Table 3).

**Table 3:** Biochemical characteristics in patients with high or low PTX3 levels.

**Parameters**	**High PTX3 concentration (≥5.9 ng/ml)**	**Low PTX3 concentration (<5.9 ng/ml)**	**t/x^**2**^**	***p*-value**
	***N =* 105**	***N* = 101**		
Gender, male (%)	63.8	58.4	0.631	0.476
Age, years	63.06 ± 7.51	63.43 ± 10.17	−0.295	0.768
Family history of cerebrovascular disease (%)	46.7	36.6	2.131	0.159
Smoking (%)	50.5	45.6	3.616	0.056
Hypertension (%)	63.8	57.4	0.879	0.393
Diabetes mellitus (%)	55.2	49.6	4.045	0.057
History of statin use (%)	41.9	37.6	0.394	0.571
BMI, kg/m^2^	27.40 ± 2.97	27.13 ± 3.16	0.644	0.521
CHOL, mmol/l	6.12 ± 0.74	6.06 ± 0.93	0.512	0.609
LDL-C, mmol/l	3.04 ± 0.74	2.57 ± 0.63	3.918	0.013^*^
TG, mmol/l	1.94 ± 0.82	1.73 ± 0.75	1.871	0.063
HDL-C, mmol/l	1.18 ± 0.31	1.20 ± 0.61	−0.227	0.821
hs-CRP, mg/l	2.99 ± 1.13	2.92 ± 1.05	0.440	0.661
TNF-α, pg/ml	1.47 ± 0.24	1.36 ± 0.21	3.586	0.018^*^

*BMI, body mass index; CHOL, total cholesterol; LDL-C, low-density lipoprotein cholesterol; TG, triglyceride; HDL-C, high density lipoprotein cholesterol; hs-CRP, hypersensitivity C-reactive protein; TNF-α, tumor necrosis factor-alpha; PTX3, pentraxin 3*.

* *P < 0.05*.

**Figure 1:**
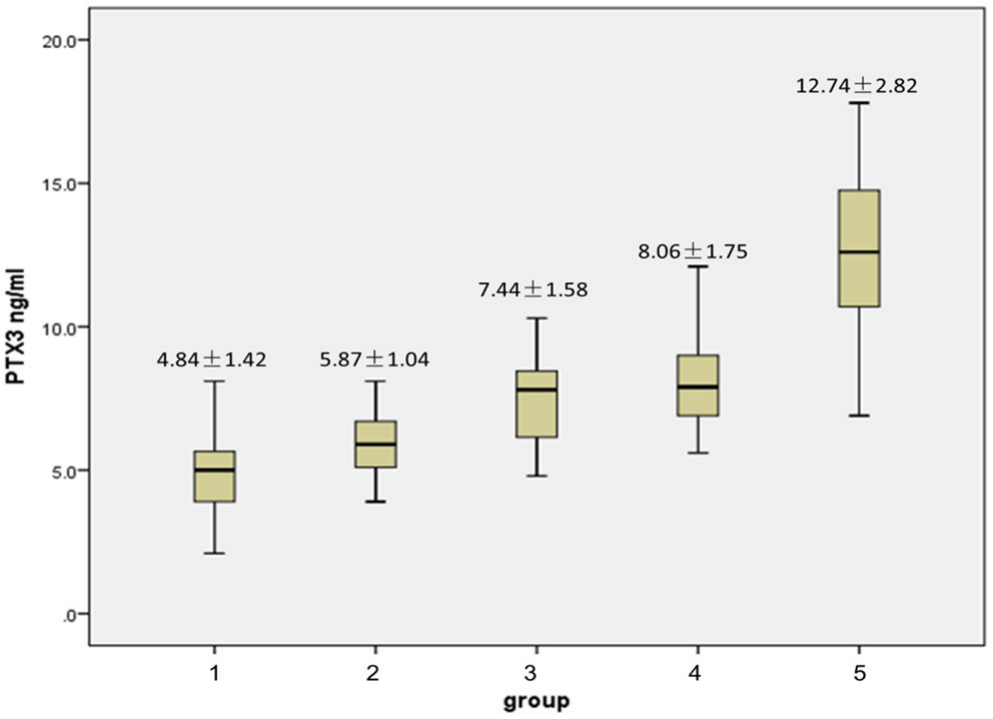
Comparison of plasma PTX3 levels between the control group and each stenosis subgroup.

Patients were divided into 5 groups based on the severity of stenosis where group 1 had no stenosis, group 2 had a single vessel with mild stenosis, group 3 had bilateral vessels with mild stenosis, group 4 had a single vessel with severe stenosis, and group 5, had bilateral vessels with severe stenosis. Levels of PTX3 in groups 2 through 5 were significantly elevated vs. group 1. Figure 1 highlights significant intra-group differences, including those between group 2 and 3, group 2 and 4, group 2 and 5, group 3 and 5, and group 4 and 5 (*p* = 0.016, 0.014, 0.000, 0.002, 0.002, respectively). However, no significance was found between groups 3 and 4 (*p* = 0.140).

### Association of PTX3 Level With Other Patient Characteristics

Logistic binary regression analysis was used to study risk factors for the presence of carotid stenosis (CAS vs. CAS-free) and severity of stenosis (SS vs. MS).

The multivariate logistic binary regression analysis revealed that increased age, elevated levels of PTX3, LDL-C, and TNF-α were all independent risk factors for occurrence of carotid stenosis. Gender, smoking history, family history of cerebrovascular disease, hypertension, diabetes mellitus, history of statin use, BMI, level of Chol, TG, HDL, and hs-CRP were not considered risk factors (Table 4).

**Table 4:** Independent risk factors for occurrence of carotid stenosis.

	**B**	**SE**	***p***	**CI 95%**	
				**Low**	**High**
Age	1.082	0.027	0.003	1.027	1.141
LDL-C	5.349	0.398	<0.001	2.450	11.677
PTX3	3.083	0.202	<0.001	2.076	4.580
TNF-α	5.466	1.310	<0.001	1.887	11.132

*LDL-C, low-density lipoprotein cholesterol; PTX3, pentraxin 3; TNF-α, tumor necrosis factor-alpha*.

In Table 5, multivariate logistic binary regression analysis of the patients with carotid stenosis (*N* = 107) revealed that high levels of PTX3 and TNF-α were independent risk factors for occurrence of severe carotid stenosis.

**Table 5:** Independent risk factors for severe carotid stenosis within stenotic patients.

	**B**	**SE**	***p***	**95%**	
				**Low**	**High**
PTX3	2.513	0.212	<0.001	1.659	3.806
TNF-α	7.339	1.812	<0.001	1.786	18.267

*PTX3, pentraxin 3; TNF-α, tumor necrosis factor-alpha*.

Multivariate linear regression was performed to study the interaction of PTX3 with age, gender, smoking history, family history of cerebrovascular disease, BMI, level of TNF-α, CHOL, TG, HDL, hs-CRP, hypertension, diabetes mellitus, and history of statin use. Only LDL-C and TNF-α levels were significantly related to PTX3 (*P* < 0.001) (Table 6).

**Table 6:** Multivariate stepwise linear regression analysis of PTX3 as a dependent variable with other factors.

	**B**	**SE**	**Beta**	***p***	**95.0%CI**	
					**Low**	**High**
LDL-C	1.490	0.228	0.399	<0.001	1.040	1.940
TNF-α	3.266	0.713	0.280	<0.001	1.861	4.671

*LDL-C, low-density lipoprotein cholesterol; TNF-α, tumor necrosis factor-alpha*.

*R = 0.544*.

Linear regression was performed to study the potential association of PTX3 level and severity of carotid stenosis. Patients were divided into the 5 groups described previously. The result revealed that PTX3 level correlated with the severity of carotid stenosis (*r* = 0.693, [95%CI, 1.965–2.623], *P* < 0.001).

## Discussion

In this study, We found that plasma levels of PTX3, TNF-α, and LDL-C were significantly higher in the CAS group than the CAS-free group, Within the CAS group, these three indicators were also significantly elevated in severe stenosis group compared to mild stenosis group. Increased age, elevated levels of PTX3, LDL-C, and TNF-α were all independent risk factors for occurrence of carotid stenosis. LDL-C and TNF-α levels were significantly related to PTX3. PTX3 was found to be tightly associated with the severity of carotid stenosis.

Prior work has shown that PTX3 is an inflammatory marker associated with coronary artery disease (10). In cerebrovascular diseases, PTX3 represents baseline atherosclerotic burden more accurately in acute stroke and is related to the severity of the stroke (11). In order to reflect the level of PTX3 more timely and accurately, we detected the concentration of PTX3 within 48 h after ischemic stroke. Ryu et al. has shown that PTX3 might be an independent prognostic marker in ischemic stroke (12). In our study, PTX3 was a more powerful predictor than traditional risk factors, including smoking, hypertension, diabetes mellitus, statin use and high density lipoprotein for carotid artery stenosis with ischemic stroke, based on logistic binary regression analysis. To our knowledge, there is no more study that showed the relationship of PTX3 levels with the occurrence and severity of carotid artery stenosis in patients with ischemic stroke.

Inflammatory mechanisms are known to play a central role in the pathogenesis and progression of atherosclerosis. Previous studies have reported that inflammatory markers such as TNF-α and IL-1βwere increased in atherosclerosis. PTX3 is stored in neutrophil granules on areas of atherosclerosis and released when neutrophils are exposed to TNF-α (13, 14). TNF-α is an inflammatory factor produced by macrophages that can exhibit inflammatory function. In our study, both levels of TNF-α and PTX3 increased significantly in the CAS group, and TNF-α increased in the PTX3 high group. Both TNF-α and PTX3 were independent risk factors for occurrence and severity of CAS. The severity of CAS was found to be correlated with PTX3 and TNF-α levels, which in turn might reflect the degree of inflammation, and may give predictive information about long term outcome in patients with ischemic stroke.

It is unclear whether high levels of PTX3 are a protective factor against atherosclerosis or a risk factor associated with the inflammatory process of atherogenesis. To further elucidate the role of PTX3 in arterial stenosis, we divided PTX3 into a high and low PTX3 group according to the median PTX3 level. The levels of LDL-C and TNF-α were significantly higher in the PTX3 high group vs. the low group. However, hypertension, diabetes mellitus, smoking, family history of cerebrovascular disease, HDL-C, and TG had no association with the concentration of PTX3. Regression analysis revealed that both LDL-C and TNF-α were significantly related to PTX3 levels, and PTX3 correlated with the severity of carotid stenosis. We have previously reported significantly higher PTX3 expression in aortic vessel walls of apolipoprotein E knockout mice and in aortic smooth muscle cells when incubated with oxidized low density lipoprotein (ox-LDL) (15). This work implicates PTX3 levels correlated with local inflammation and the formation of atherosclerosis, and illustrates its association with lipid metabolism. Prior studies have established a relationship between PTX3 level and tissue damage. Persistently elevated PTX3 levels may be associated with morbidity and severity of cardiovascular diseases, and positively associated with risk factors for cardiovascular disease, including low levels of HDL-C, arterial stiffness, and obesity (16). In a mouse model of superior mesenteric artery ischemia and reperfusion, PTX3 levels correlated with local and remote inflammation and tissue injury. PTX3 also induced mouse endothelial cell inflammation and dysfunction. It is also reported that PTX3 overexpression can worsen ventricular dysfunction and increase tissue damage, hypertrophic responses, and ventricular dysfunction in a murine cardiac injury model (17, 18). Whether the role of PTX3 in carotid arterial stenosis is beneficial or harmful may require further animal and cell experiments and more sample sizes to further clarify.

The correlation between PTX3 and lipid metabolism has already been attended in recently. PTX3 and LDL-C have been shown to correlate with severity of coronary artery disease in patients with intermediate to high atherosclerosis severity (19). In this study, multivariate stepwise linear regression analysis showed that among LDL, TG, HDL, and total cholesterol, only LDL levels significantly correlated with PTX3 level. This correlation between LDL and PTX3 suggests a mechanism connecting LDL to inflammation mediators. High levels of PTX3 have been found in plasma from patients with extensive atherosclerosis and increased LDL cholesterol (20–22). In patients with hypercholesterolemia, PTX3 correlated with the severity of vascular disease and statin therapy decreased plasma PTX3 levels (22), suggesting the involvement of PTX3 in the mechanisms by which LDL cholesterol triggers vascular inflammation. Higher plasma PTX3 levels were found in familial hypercholesterolemia (FH) patients, and statin treatment again reduced the PTX3 level in these patients (23). A significant increase of PTX3 mRNA levels have also been found in adipose tissue of patients with high levels of LDL.

## Limitation

One limitation of this study is the number of patients included. It would be better to include more patients to credibility of the results. In addition to that, there was no long-term follow-up of the patients, and did not provide any prognostic data of the CAS. The ischemic stroke is a complex disease. In addition to carotid artery, vertebral arteries and basilar artery, play important role in ischemic stroke, as well as intracranial arteries. If more patients were included and more investigations on vertebral artery and basilar artery were performed as well, we might have systemic assessment of the role of PTX3 in ischemic stroke.

## Conclusions

High plasma PTX3 levels, TNF-α, and LDL-C are significantly correlated with the prevalence and severity of carotid artery stenosis. PTX3 may be a more powerful predictor for the severity of carotid artery stenosis.

## Data Availability Statement

All datasets generated for this study are included in the article/supplementary material.

## Ethics Statement

The studies involving human participants were reviewed and approved by Bioethics Committee of Beijing Friendship Hospital, Capital Medical University (2019-P2-063-01). The patients/participants provided their written informed consent to participate in this study.

## Author Contributions

LY and JT participated in study concept or design. TZ and JL collected and analyzed the CTA data. LY and CS examined the blood sample and performed the statistical analyses. LY, FG, and WZ collected and analyzed the patients and blood data.

### Conflict of Interest

The authors declare that the research was conducted in the absence of any commercial or financial relationships that could be construed as a potential conflict of interest.
